# Regulatory Network of Cotton Genes in Response to Salt, Drought and Wilt Diseases (*Verticillium* and *Fusarium*): Progress and Perspective

**DOI:** 10.3389/fpls.2021.759245

**Published:** 2021-11-29

**Authors:** Masum Billah, Fuguang Li, Zhaoen Yang

**Affiliations:** ^1^State Key Laboratory of Cotton Biology, Institute of Cotton Research, Chinese Academy of Agricultural Sciences, Anyang, China; ^2^Zhengzhou Research Base, State Key Laboratory of Cotton Biology, Zhengzhou University, Zhengzhou, China

**Keywords:** cotton, genes, network, drought, salt, *Verticillium*, *Fusarium* wilt

## Abstract

In environmental conditions, crop plants are extremely affected by multiple abiotic stresses including salinity, drought, heat, and cold, as well as several biotic stresses such as pests and pathogens. However, salinity, drought, and wilt diseases (e.g., *Fusarium* and *Verticillium*) are considered the most destructive environmental stresses to cotton plants. These cause severe growth interruption and yield loss of cotton. Since cotton crops are central contributors to total worldwide fiber production, and also important for oilseed crops, it is essential to improve stress tolerant cultivars to secure future sustainable crop production under adverse environments. Plants have evolved complex mechanisms to respond and acclimate to adverse stress conditions at both physiological and molecular levels. Recent progresses in molecular genetics have delivered new insights into the regulatory network system of plant genes, which generally includes defense of cell membranes and proteins, signaling cascades and transcriptional control, and ion uptake and transport and their relevant biochemical pathways and signal factors. In this review, we mainly summarize recent progress concerning several resistance-related genes of cotton plants in response to abiotic (salt and drought) and biotic (*Fusarium* and *Verticillium* wilt) stresses and classify them according to their molecular functions to better understand the genetic network. Moreover, this review proposes that studies of stress related genes will advance the security of cotton yield and production under a changing climate and that these genes should be incorporated in the development of cotton tolerant to salt, drought, and fungal wilt diseases (*Verticillium* and *Fusarium*).

## Introduction

Cotton (*Gossypium* spp.) is one of the most important cellulosic fiber crops, contributing about 35% of total fiber worldwide and also important for oilseed crops. Cotton crops grow well in in the tropics and subtropics in over 80 countries, and cotton is considered the leading crop in about 30 of these countries (Abdelraheem et al., 2019). China is the top cotton fiber producing country. Approximately, 2/3 of cotton fiber derives from China, India, the United States, Pakistan, and Brazil (Khan et al., 2020). According to prediction models for the next 50–100 years, surface temperatures will increase by 3–5°C, radically disturbing agricultural systems worldwide (Solomon et al., 2007). Rising temperatures will increase the frequency of drought, flood, and soil salinization areas, and decrease cultivable land for agriculture. Drought alone is currently reported to affect about 45% of agricultural land worldwide; likewise, about 19.5% of the cultivable agricultural lands are under salinity stress (Dos Reis et al., 2012). Ullah et al. (2017) reported that cotton fiber production affected by drought and heat stress may lead to yield loss of about 34%. Drought and salinity combined may reduce >50% of arable land on average in the next 20 years (Abdelraheem et al., 2019). In addition, crop plants are subjected to various pests and pathogens, such as fungi, viruses, bacteria, nematodes, and herbivorous insects. *Fusarium* and *Verticillium* wilt diseases caused by soil-borne fungal pathogens of cotton plants consistently cause extreme yield losses in cotton producing countries including China (Cun et al., 2002; Davis et al., 2006). These two diseases were likely introduced into China in the 1930s, and expanded throughout the main cotton planting areas by the 1970s (Li X. et al., 2017). In the early 1980s, these diseases caused serious problems for cotton production, resulting in >150 thousand tons of lint cotton per year in China (Chen et al., 1985). At present, these two wilt diseases are considered the main impediments for producing quality cotton with sustainable yields in China (Pei et al., 2020).

**FIGURE 1:**
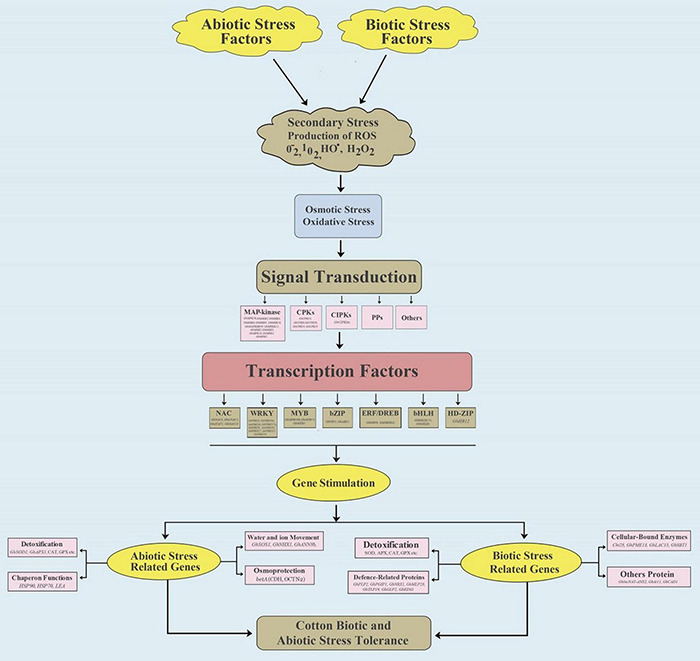
Overall representation of cotton gene regulatory networks in response to abiotic (Salt and Drought) and biotic (*Verticillium* and *Fusarium*) stresses. In the MAP-kinase signaling, abiotic stress genes include *GhMAPKKK49*, *GhMEKK12*, *GhMKK1*, *GhMKK3*, *GhMPK16*, *GhMPK3*, and *GbMPK3*, while biotic stress genes include *GhMPK20*, *GhMKK10*, *GhMKK9*, *GhMKK6*, *GhMKK4*, and *GhMKK2*. In the CPKs family, *GhCPK8*, *GhCPK38*, *GhCPK54*, and *GhCPK55* are involved in abiotic stress, and *GhCPK33* is involved in biotic stress. In the CIPKs family, *GhCIPK6a* is involved in abiotic stress. In the transcriptional factors, for abiotic and biotic stress, NAC includes *GhirNAC2*, *GhATAF1*, *GhNAC18*, and *GbNAC1*, MYB includes *GhMYB73*, *GbMYB5*, *GhMYB108*, and *GhMYB108*, WRKY includes *GhWRKY46*, *GhWRKY41*, *GhWRKY27a*, *GhWRKY6*, *GhWRKY91*, *GhWRKY17*, *GhWRKY25*, *GhWRKY33*, and *GbWRKY1*, bZIB includes *GhABF2* and *GbVIP1*, ERF/DREB includes *GhERF2*, *GhDREB1L*, and *GbERFb*, and bHLH includes *GhbHLH1* and *GbbHLH171*. HD-ZIP includes *GhHB12* for biotic stress.

Cotton plants are exposed to combinations of stress factors in all growth environments. Each stress factor stimulates a complex cellular and molecular network in the crop plants to avoid injury and provide defense, while preserving growth and production (Herms and Mattson, 1992) (Figure 1). To better understand plant responses, Mittler and colleagues proposed a “stress matrix” to identify the complex interactions among multiple abiotic and biotic stresses (Mittler, 2006; Suzuki et al., 2014; Pandey et al., 2017). Stress tolerance genes are activated by a variety of factors such as salinity, drought, heat, cold, and active oxygen balance, and they include membrane permeability, hormone signal transduction, and osmotic regulation (Noctor et al., 2014; Tian et al., 2015). Crop plants trigger a definite and distinctive stress mechanism when exposed to combined stresses (Rizhsky et al., 2004). Multiple stress factors result in overproduction of reactive oxygen species (ROS), such as H_2_O_2_, causing widespread cell damage and suppressing photosynthesis. Generally, crop plants use a complex antioxidative defense system to repair or prevent damage through exciting multiple stress-related genes (Ciarmiello et al., 2011) (Figure 1). Genes associated with the antioxidative defense system are divided into three major groups: (1) genes that participate directly in the defense of cell membranes and proteins, such as late embryogenesis abundant (LEA) proteins, heat shock proteins (HSPs) or chaperones, antifreeze proteins, osmoprotectants, free-radical scavengers, and detoxification enzymes (Wang and Jiao, 2000; Vinocur and Altman, 2005); (2) genes intricate in signaling cascades and transcriptional control, e.g., mitogen-activated protein kinase (MAPK), phospholipases, calcium-dependent protein kinase (CDPK), SOS kinase, phospholipases, and transcription factors (TFs) (Frank et al., 2000; Wang et al., 2003; Ludwig et al., 2004; Vinocur and Altman, 2005); and (3) genes that participate in ion uptake and transport (Vinocur and Altman, 2005).

It is necessary to identify and characterize stress-inducible genes to understand molecular functions as well as generate stress tolerant crops through gene manipulation. In cotton, several genes and gene families have been identified, related to salt stress (Yao et al., 2011; Dongdong et al., 2016; He et al., 2016; Liang et al., 2016; Gao et al., 2018), drought stress (Luo et al., 2013; Dongdong et al., 2016; Liang et al., 2016; Li F. et al., 2017; Wang W. et al., 2019), and wilt diseases of *Fusarium* (Wang et al., 2009, 2010; Liu N. et al., 2017; Pei et al., 2020; Wang C. et al., 2020) and *Verticillium* (Li et al., 2014; Xu et al., 2016; Tang et al., 2019; Feng et al., 2021), but their molecular mechanisms are still unknown. Our understanding of advanced molecular mechanisms and signaling pathways of genes in response to biotic and abiotic stresses is still limited, but recent gene discoveries provide a foundation for future research. In this review, we mainly summarize recent studies of cotton genes differentiated according to their molecular functions in response to salt, drought, and *Fusarium* and *Verticillium* wilt diseases. The objective of this review is to update knowledge about regulation of cotton genes in response to salt, drought, and wilt diseases and describe recent advances in these stress-response mechanisms.

## Genes Involved in Response to Salt and Drought Stress

### Mitogen-Activated Protein Kinases

Mitogen-activated protein kinase cascades are evolutionarily preserved signal transduction pathways involved in transducing extra-cellular cues to the nucleus for proper cellular regulation through phosphorylation of downstream signaling marks into eukaryotic cells. MAPK cascades are divided into three kinases: mitogen activated protein (MAP) kinase kinase kinases (MAPKKKs), MAP kinase kinases (MKKs), and MAPKs. MAPKs are located in the cytoplasm and nucleus and are involved in various cellular processes like growth, development, and multiple stress stimulus (Danquah et al., 2015; Wang et al., 2015). MAPK cascades play multiple roles as both positive and negative regulators in environmental stress (Lin et al., 2021).

In upland cotton (*Gossypium hirsutum*), 52 GhMAPKs, 23 GhMAPKKs, 166 GhMAPKKKs, and in *G. raimondii* 28 putative MAPK cascade genes, were identified (Zhang X. et al., 2014; Yin et al., 2021), while very few genes were characterized over the salt and drought stresses.

Dongdong et al. (2016) suggested that cotton *GhMAPKKK49* responds to multiple external stresses and may be involved in jasmonic acid (JA), ethyl- ene (ET), salicylic acid (SA), abscisic acid (ABA) and H_2_O_2_-mediated signaling pathways. Gene-silenced *GhRAF4* and *GhMEKK1*2 cotton plants exhibited decreased drought tolerance by the rapid accumulation of malondialdehyde (MDA), superoxide dismutase (SOD), and peroxidase (POD) (Zhang et al., 2020). Conversely, *GhRaf19*, a Raf-like MAPKKK gene, controls cotton plant tolerance to drought and salt by reducing cellular ROS (Jia et al., 2016). MAP kinase cascade is reported to phosphorylate and stimulate a key WRKY TF, exposing a regulation module, including *GhMAP3K15-GhMKK4-GhMPK6-GhWRKY59-GhDREB2*, that has a role in modulating cotton drought resistance (Li F. et al., 2017). *GhMKK1* was highly induced by treatment of salt, drought, and H_2_O_2_, whereas Overexpression of *GhMKK1* in tobacco improved resistance to salinity and drought, which was determined by the regulation of ROS scavenging capability (Lu et al., 2013). Silencing *GhMKK3* in cotton resulted in susceptibility to drought stress. In contrast, overexpression of *GhMKK3* in *N. benthamiana* initiated drought resistance by contributing to regulation of ABA-induced stomatal closure and root hair growth (Wang C. et al., 2016).

In addition, *GhMKK3* and *GhPIP1* act together with *GhMPK7* to regulate drought and ABA-activated MAPK elements (Danquah et al., 2015). Therefore, *GhMKK5* had a negative role in response to salt and drought stress in transgenic tobacco (Zhang L. et al., 2012). *GhMPK16* might be involved in several signal transduction pathways, participating in both biotic and abiotic stress signaling pathways. *GhMPK16* exhibited significant resistance to fungi and bacteria in transgenic *Arabidopsis*, but resulted in sensitivity to drought tolerance and rapid H_2_O_2_ accumulation (Shi et al., 2011). In overexpressing transgenic *Arabidopsis*, *GhMPK17* was found to contribute in the plant response to high salinity and osmotic stresses and ABA-mediated signaling pathways (Zhang J. et al., 2014). *GbMPK3* may also be a positive regulator of drought tolerance through regulating ROS (Long et al., 2014). Recently, *GhMPK3* was identified and characterized from upland cotton. Silencing *GhMPK3* increased drought tolerance in cotton plants, whereas overexpression improved plant resistance to drought, cold, and salt stress (Sadau et al., 2021). These findings help in better understanding of the regulatory network of MAPKs under salt and drought stresses, and offer another strategy for improving stress tolerance in cotton crop production.

### Transcription Factors

Transcription factors are crucial in gene expression of plants, including stress-response, hormones, cell division, and organ development. Of the more than 80 TF families, only NAC, MYB, apetala2/ethylene responsive factor (AP2/ERF), basic leucine zipper (bZIP), basic helix-loop-helix (bHLH), WRKY families have been studied to understand their roles in response to salt and drought stresses. Transcription factors either negatively or positively control gene expression, which determines plant survival under environmental stress (Ijaz et al., 2020). Therefore, in order to comprehend the mechanism of stress tolerance, it is critical to investigate the transcription factors involved in regulating gene expression. Few reports about TF genes in cotton have been published but results of available studies have shown that FTs plays an important role in responding to cotton salinity and drought (Evans et al., 2016; Baillo et al., 2019; Debbarma et al., 2019; Li Z. et al., 2019; Zhao et al., 2019).

In total, 495 NAC genes were identified in three cotton species and the evolution and diversity of these genes was explored in cotton plants (Fan et al., 2018). The expression patterns, co-expression network, and transactivation of *GhNAC* were studied in response to salt and drought stresses (Sun et al., 2018). Overexpression of *SNAC1* has a significant role in drought and salt tolerance in cotton through improving root development and decreasing transpiration rates (Liu G. et al., 2014). Both gain and loss of function studies revealed that ABA inducible *GhirNAC2* has a positive role in cotton drought tolerance *via* regulating stomata closure and *GhNCED3a/3c* expression (Shang et al., 2020). Moreover, *GhATAF1*, a stress-responsive NAC TF, functions directly in the response to salinity stress with the activation of SA-mediated signaling but suppression of JA-mediated signaling (He et al., 2016). Evans et al. (2016) reported that *GhNAC18* was induced in leaf senescence by treatment with various phytohormones including methyl jasmonate (MeJA), salicylic acid (SA), and ethylene (ET) but was down-regulated by abscisic acid (ABA). In addition, *GhNAC18* was upregulated by drought but suppressed by high salinity stress.

Myeloblastosis (MYB) TFs are extensive and considered the most functionally diverse gene family of all TFs in plants. MYB TFs work as dynamic regulators, modulating the response of abiotic stress in crop plants. A total of 524 MYB TF encoding genes in *G. hirsutum* and 205 putative R2R3-MYB genes in *G. raimondii* have been identified (Salih et al., 2016). However, much less is known about MYB proteins in cotton in response to drought and salt stresses.

*GrMYB020, GrMYB074, GrMYB163, GrMYB170*, and *GrMYB201* exhibited significant increasing patterns under stresses of drought and/or salt, while *GrMYB121, GrMYB169, GrMYB 176, GrMYB188*, and *GrMYB190* were induced in response to salt and drought treatment (Dongdong et al., 2016). *GhMYB73* clearly improved tolerance to salt and ABA stress in transgenic *Arabidopsis* (Zhao et al., 2019). *GbMYB5* decreased plant water loss capability by regulating the expression of dehydration-responsive genes in the ABA-mediated signaling pathway, sustained the maintenance of plant cells and proteins by activating biosynthesis of osmolytes and LEA proteins, and efficiently detoxified ROS (Chen T. et al., 2015). Moreover, various physico-chemical characteristics of the *GhMYB108*-like gene have been described, suggesting that *GhMYB108*-like is a crucial regulatory gene under drought and salinity stresses (Ullah et al., 2020).

WRKY proteins are about 60 amino acids long and contain one or two highly conserved WRKYGQK motifs as well as a typical zinc-finger structure. They can identify and bind to W-box cis-regulatory elements. WRKY is large TF family of transcriptional regulators, whose members are involved in diverse processes in plants responding to both biotic and abiotic stress. WRKY family genes function in ROS regulation and in mitigating the adverse effects of oxidative stress in cotton and can have positive or negative roles in response to salt and drought stresses. A total of 102, 112, and 109 WRKY genes were, respectively identified in *G. hirsutum*, *G. raimondii*, and *G. arboreum* and their functions were also characterized (Dou et al., 2014; Ding et al., 2015). Overexpression of *GhWRKY46* in *Arabidopsis* improved tolerance to salt and drought by enhancing survival rates, chlorophyll content, and biomass content (Li Y. et al., 2021). Moreover, the constitutive expression of *GhWRKY41* in transgenic tobacco plants advances salt and drought tolerance by regulating stomatal closure in ABA-mediated pathways (Chu et al., 2015). Overexpression of *GhWRKY27a* reduces the drought tolerance of transgenic tobacco plants by enhancing stomatal opening and attenuating expression of ABA (Yan et al., 2015).

Recently, Li Z. et al. (2019) reported that *GhWRKY6* overexpression in *Arabidopsis* enhanced salt and drought sensitivity by regulating stomatal aperture, enriching ROS, reducing proline content, and increasing electrolyte and MDA contents in ABA signaling pathways. A transient dual-luciferase reporter method confirmed that *GhWRKY91* stimulated the expression of *GhWRKY17* and negatively regulated natural and stress-induced leaf senescence with ABA signals and ROS production (Gu et al., 2019). Overexpression of *GhWRKY25* in tobacco decreased the plant’s resistance to drought stress, but improves resistance to salt stress, suggesting that this gene has both positive and negative functions in response to abiotic stresses (Liu et al., 2016). Under drought stress, *GhWRKY33* overexpressing transgenic *Arabidopsis* plants were emaciated much more quickly than wild-type plants because of earlier water loss. Additionally, *GhWRKY33* transgenic plants exhibited more tolerance in ABA-mediated media (Wang N.N. et al., 2019).

Basic leucine zipper TF family genes have significant roles in diverse biological processes in response to biotic and abiotic stresses (Alves et al., 2013; Sornaraj et al., 2016). Some bZIP genes have been characterized in crops other than cotton in response to salt and drought stresses. A total of 228 bZIP genes in *G. hirsutum*, 91 in *G. arboreum*, and 86 in *G. raimondii* have been identified (Khanale et al., 2020). GHbZIPs involved in endoplasmic reticulum (ER) stress were supposed to stimulate abiotic stress signals through interaction with other GHbZIPs (Wang et al., 2020b). *GhABF2*, a bZIP TF significantly enhanced salt and drought stress tolerance in transgenic *Arabidopsis* and cotton plants, while suppression of GhABF2 modulated transgenic cotton sensitive to drought and salinity stress. Moreover, *GhABF2* expression was induced by ABA treatment but was inhibited by high salinity (Liang et al., 2016). *ABP9* is another maize bZIP encoding gene that improves salt and drought tolerance by altering physiological and biochemical processes as well as stress-related gene expression, and it may induce the ABA signaling in transgenic cotton (Wang C. et al., 2017).

Ethylene-responsive factor/dehydration-responsive element-binding (ERF/DREB) proteins are in the AP2/ERF (APETALA 2/ethylene-responsive element binding factor) TF family and make up a large TF subfamily, which was first documented in *Arabidopsis* (Jofuku et al., 1994; Sakuma et al., 2002). The ERF/DREB subfamily contains stress-responsive factors, and several of these genes are participated in both biotic and abiotic stress responses (Zhang et al., 2013). ERF/DREB TFs are essential in ABA-independent signaling pathways, which modulate stress-induced genes (Baillo et al., 2019), to form an inter-connected stress controlling network. Many AP2/ERF genes respond to plant stress hormones such as ABA and to help regulate ABA and ET dependent and independent stress responsive genes (Xie et al., 2019). However, a total of 504 AP2/EREBPs in *G. hirsutum* and 269 AP2/EREBP genes in *G. raimondii* (D5) were identified through a genome wide association study (GWAS) (Liu and Zhang, 2017), and their functions in response to abiotic stress in cotton were proposed.

The novel cotton gene *GhDREB1L* might have a significant role in response to drought and high salinity through binding to the DRE *cis*-element (Huang et al., 2007). RNA blot evaluation confirmed that the *GhDREB* gene was induced by high salt, drought, and cold stresses in cotton seedlings. Similarly, *GhDREB* in transgenic wheat confers promoted tolerance to high salt, drought, and freezing stresses (Gao et al., 2009). Overexpression of *StDREB2* in cotton might improve drought tolerance by up-regulating *GhERF2*, *GhDREB1B*, *GhDREB1A*, and antioxidant genes (Baillo et al., 2019). *GhDREB40D* and *GhDREB7A* from *G. hirsutum* have a positive role in responding to drought stress in *G. herbaceum*. Moreover, *GhERF38* acts as a novel regulator and is involved in response to salt and drought stress and ABA signaling by regulating stomatal aperture of guard cells during plant development (Ma et al., 2017).

The basic helix-loop-helix (bHLH) is a functionally diverse group of TFs found in both plants and animals. bHLH TFs have been demonstrated to contribute in regulating several abiotic stresses in plants (Khan et al., 2018), though very little is known about cotton bHLH proteins. Expression of *GhbHLH1* in leaves was rapidly induced by ABA and drought (PEG) treatments, suggesting that bHLH may function as a regulator of ABA signaling and drought stress in cotton (Meng et al., 2009). However, these findings suggest that transcriptional regulation of stress-responsive genes is an important step in determining the mechanisms underlying salt and drought stress responses, and that these transcription factors may be key targets for the development of cotton crops with enhanced salt and drought stress tolerance.

### Reactive Oxygen Species-Scavenging and Detoxification Proteins

The antioxidant system is central to maintaining cell activity in plants by detoxifying ROS under stress conditions. Plants evolved antioxidant defense systems that can not only detoxify ROS but also adjust ROS levels required for proper cell signaling (Gupta et al., 2019). Here we discuss genes involved in enzymatic and non-enzymatic systems for ROS scavenging that regulate the state of detoxification and homeostasis in plant cells in response to salt and drought conditions. These genes annex ascorbate peroxidase (APX), catalase (CAT), glutathione reductase (GR), glutathione peroxidase (GPX), glutathione S-transferase (GST), monodehydroascorbate reductase (MDHAR), dehydroascorbate reductase (DHAR), myo-inositol monooxygenease (MIOX), peroxiredoxin (PRX), proline synthesis, and superoxide dismutase (SOD). The information regarding their functions and regulatory mechanisms in cotton are limited.

Ascorbate peroxidase is required for the first step of the AsA-GSH cycle, which scavenges ROS and protects the plant cell from stress damage (Prashanth et al., 2008). A recent study investigated the role of APX in protecting cellular oxidative homeostasis of stomatal guard cells and in regulating cotton photosynthesis (Guo et al., 2020). In allotetraploid cotton, a total of 26 APX genes were found (Tao et al., 2018). *GhAPX8/9/10* is a new *APX* gene that is not found in rice and *Arabidopsis*, but its molecular function is still unknown. APX-silenced cotton fibers displayed more sensitivity to oxidative stress than wild-type plants, and the overexpression of *GhAPX1* enhanced tolerance of fibers to oxidative stress in cotton (Guo K. et al., 2016). Therefore, simultaneously overexpressing *GhSOD1* and *GhAPX1* showed no effect to methyl viologen and salt stress (Luo et al., 2013).

Catalases gene family members are considered to be great ROS-scavenging proteins associated with various physiological functions in plant growth, development, and stress responses (Wang W. et al., 2019). As noted for other genes, there is very little known about CAT genes in cotton. A total of seven CAT genes have been identified in the genomes of *G. hirsutum* and *G. barbadense* (Wang W. et al., 2019).

Superoxide dismutases mainly convert highly reactive superoxide radicals into hydrogen peroxide and molecular oxygen and are associated with a group of proteins that play an important role in the stress response of plants (Wang et al., 2016a). SODs are designated as the frontline defence within the plant systems against ROS and are categorized by the metal ions that are bound to their active sites such as iron (FeSOD), copper/zinc (Cu/ZnSOD), and manganese (MnSOD). A total of 18 SOD genes have been identified from *G. hirsutum*, *G. raimondii*, and *G. arboreum* (Wang et al., 2016a; Wang W. et al., 2017). In cotton, a previous study revealed that overexpression of SODs improved tolerance to salt stress and oxidizing stress induced by methyl viologen, showing that SODs increased cotton resistance to abiotic stress (Luo et al., 2013).

Peroxidases are involved in various plant physiological systems, including cell elongation, cross-linking of cell wall components, auxin metabolism, lignin and suberin formation, phytoalexin synthesis, defense against biotic or abiotic stress, and metabolism of reactive nitrogen species and ROS from germination to senescence (Marjamaa et al., 2006; Almagro et al., 2009). Duan et al. (2019) identified 198 non-redundant *GhPOD* genes. A recent expression study on cotton POD genes proposed that they are crucial under high salt stress (Li S. et al., 2020).

The plant glutathione peroxidase (GPX) family includes multiple isoenzymes and has a significant role in ROS hemostasis by catalyzing the decline of H_2_O_2_ and other organic hydro-peroxides to defend plant cells under environmental stress responses. A total of 13 putative GPXs from the genome of *G. hirsutum* (*GhGPXs*) were identified and the expression patterns of GhGPX transcripts were observed under short-term exposure to salt, osmotic, and ABA-induced stresses to understand their role in these stresses. Additionally, in terms of their role under abiotic stresses, gpx3Δ (H_2_O_2_-sensitive mutant) of *Saccharomyces cerevisiae* was complemented with the *GhGPXs*, revealing their participation in the oxidative stress response (Chen M. et al., 2017). There are no reports available that provide insights on expression profiling or functional validation of GPXs in cotton under salt and drought stresses.

Glutathione S-transferases are ancient and ubiquitous proteins that are part of a large gene family and have great versatility in organisms. Based on gene association and amino acid sequence the plant GSTs can be divided into four classes, including Phi (F), Tau (U), Lambda (L), and dehydroascorbate reductase (DHAR) (Dong et al., 2016). GST together with Glutathione (GSH) can decrease Peroxidase POX activity through scavenging in the cell. When plants are subjected to abiotic stress, this enzyme is highly induced (Nadarajah, 2020). Over 100 GST genes from maize, soybean, and Arabidopsis have been identified (Pandey P. et al., 2015), with multiple functions such as apoptosis, cellular metabolism, hormone homeostasis, cellular detoxification, and responses to various other biotic and abiotic stresses (Wang L. et al., 2016), but in cotton their roles are limited. Transcriptome analysis of 40 selected GST genes showed tissue-specific expression patterns and salt stress either induced or suppressed their expression levels. These findings provide insight into the function and evolution of the GST gene family in cotton in response to salt stress (Dong et al., 2016). A GST gene (*Gst-cr1*) from cotton was introduced into tobacco plants, and overexpressing *Gst-cr1* exhibited enhanced resistance to oxidative stress induced by methyl viologen (Yu et al., 2003).

Monodehydroascorbate reductase is a key enzyme in ascorbate-glutathione recycling that regulates ascorbic acid (AsA)-mediated reduction/oxidation (redox) regulation and thus plays critical roles in plant cell growth, development, and physiological and molecular responses to environmental stress (Zhou et al., 2021). The MDHAR defensive system protects plant cells against oxidative stress damages (Gill and Tuteja, 2010). In cotton, information regarding MDHAR function and regulatory mechanisms in response to abiotic stress is limited. The identification and universal bioinformatic analysis of 36 MDHAR family genes in *G. hirsutum*, *G. arboreum*, *G. raimondii*, and *G. barbadense* were conducted. GhMDHAR expression pattern analysis in different cotton tissues, as well as under abiotic stress and phytohormone treatment, revealed a diverse of expression features (Zhou et al., 2021). These findings provide a comprehensive understanding of cotton plant antioxidant gene families and lay the foundations for decoding the molecular mechanisms of these genes in response to salt and drought stress.

### Calcium Transporters and Binding Proteins

Calcium (Ca^2+^) is a universal secondary messenger in cell signal transduction pathways that functions directly in physiological and molecular processes. Ephemeral alterations of the cytoplasmic Ca^2+^ level in response to multiple stresses are recognized and interpreted by various Ca^2+^ sensors or Ca^2+^ binding proteins, which pass the signals into downstream response processes such as stimulation of gene expression patterns and phosphorylation cascades (Tuteja and Mahajan, 2007). The regulation of gene expression by calcium is critical for plant defense against abiotic stress. Transient changes in cytoplasmic Ca^2+^ levels have been demonstrated in response to salinity, drought, cold, wounding, and pathogens (Hetherington and Brownlee, 2004). Plant Ca^2+^ binding proteins can be divided into four classes: calcium-dependent protein kinases (CDPK), calcineurin B-like proteins (CBL), calmodulins (CaM), and calmodulin-like proteins (CaML) (Cheng et al., 2002; Kolukisaoglu et al., 2004).

Among them, CDPKs are the best categorized and are of specific interest to cotton plants, which comprise a large multi-gene family and their roles in response to various stresses have been described. CDPKs from different plants have been identified, and their regulatory mechanisms in plant development or stress responses have been investigated (Nadarajah, 2020), but again, little is known about responses to salt and drought stresses in cotton plants. A total of 98 predicted CDPK genes from *G. hirsutum* and 41 from *G. raimondii* were identified (Liu W. et al., 2014; Gao et al., 2018). An earlier study proposed that CDPK gene expression in response to various abiotic stresses would be useful for identifying *GhCDPKs*, which may have important roles in cotton adaptation to abiotic stresses (Li et al., 2015). A total of 19 CPKs were identified for their rapid transcriptional responses to salt stress, the majority of which were also induced by ethephon, indicating that the salinity and ethylene responses overlapped. Moreover, silencing of four CPK genes (*GhCPK8*, *GhCPK38*, *GhCPK54*, and *GhCPK55*) severely decreased tolerance to salt stress (Gao et al., 2018), which suggested that the sensing and regulatory network of CPKs in cotton are involved in the response to salt stress. CPK11 from *Arabidopsis* has recently been shown to phosphorylate drought-induced protein 19 (GhDi19–1 and GhDi19–2) in cotton plants (Qin et al., 2016).

Calcineurin B-like protein-interacting protein kinase (CIPK) is modulated by calcineurin B-like protein (CBL) and is an important component of Ca^2+^ signal transduction with a significant role in plant abiotic stress. CIPKs are well documented to act as vital elements in plant salt and drought stress signaling pathways (Pandey G.K. et al., 2015; Luo et al., 2017). Through genome wide analysis, in *G. hirsutum* and *G. barbadense*, a total of 80 and 78 CIPK genes were identified, respectively (Cui et al., 2020). The analysis of CIPK transcriptome sequence data under abiotic stresses (drought, salt, and low temperature) in different tissues at the trefoil stage revealed that these stresses induced CIPK expression in cotton (Wang J. et al., 2016). *GhCIPK6a* overexpression lines demonstrated higher salt tolerance, which was achieved through involvement in ROS scavenging and MAPK pathways (Su et al., 2020). Thus, Ca^2+^ transporters and binding proteins have the potential to be used in the cotton regulatory network and breeding to improve stress-tolerance.

### Other Proteins in Response to Salt and Drought Stresses

Plant Na^+^/H^+^ antiporters (NHXs) are membrane transporter proteins that regulate cellular Na^+^/K^+^ and pH homeostasis levels. In plant development and salt stress response, considerable evidence has underlined the crucial roles of the NHX family (Bassil and Blumwald, 2014). Physiological and molecular evidence showed that co-overexpression of *AVP1* and *AtNHX1* in transgenic cotton further improves drought and salt tolerance (Shen et al., 2015). *GhSOS1* is a plasma membrane Na + /H + antiporter gene that improves salt tolerance in transgenic Arabidopsis by increasing the expression of stress-related marker genes, while silencing *GhSOS1* reduced cotton tolerance to salt (Chen X. et al., 2017). In a recent study, a vacuolar localized protein, *GhNHX1*, was induced by salt stress in cotton and loss of function of *GhNHX1* showed enhanced sensitivity in cotton seedlings to high salt concentrations. This finding suggests that *GhNHX1* positively regulates salt stress to cotton (Long et al., 2020). PYR/PYL/RCAR proteins are putative ABA receptors with vital roles in both plant biotic and abiotic stresses (Santiago et al., 2009).

Annexins are a multi-gene family that is highly conserved in plants, animals, and fungi (Davies, 2014) whose members mediate calcium transport and Ca^2+^ conductance in plant cells (Demidchik et al., 2018). Little is known about phosphatases that dephosphorylate annexins, especially during salinity stress-induced Ca^2+^ influx. A cotton phosphatase *GhDsPTP3a* and an annexin protein *GhANN8b* interact and conversely regulate Ca^2+^ and Na^+^ fluxes under salinity stress in cotton (Mu et al., 2019). Most of the controlling mechanisms of auxin are regulated by auxin-responsive genes, which are mainly involved in homeostasis based on catalytic activities. These genes are divided into three categories: Aux/IAA, SAUR, and GH3 (Goda et al., 2004). However, few studies on these genes in relation to environmental stresses have been conducted (Yuan et al., 2013). The functional characterization of Gh A08G1120 (GH3.5) plants using virus-induced gene silencing (VIGS) revealed that silenced plants were more sensitive to drought and salt stresses than wild types (Kirungu et al., 2019). In upland cotton, a total of 27 predicted PYL proteins were identified. Overexpression of *GhPYL10*, *GhPYL12*, and *GhPYL26* in *Arabidopsis* increase sensitivity to ABA but confer tolerance to drought stress in transgenic plants (Chen Y. et al., 2017).

Detoxification efflux carriers (DTX)/multidrug and toxic compound extrusion (MATE) transporters are important in the translocation of ABA, a phytohormone with functions in plants under multiple abiotic stresses (Tiwari et al., 2014). *Gh_D06G0281* (DTX/MATE) overexpressing *Arabidopsis* improved tolerance to salt, drought, and cold stress with a high level of antioxidant enzyme production and significantly lower levels of oxidant (Lu et al., 2019). Additionally, recent genome wide studies of PIN-FORMED (*PIN*), respiratory burst oxidase homolog (*Ghrboh*), Valine-glutamine (*VQ*) gene family, heat shock protein 20 (Hsp20), at-hook motif containing nuclear localized (*AHL*), leaf senescence, protein phosphatases (PP2C), 9-cisepoxycarotenoiddioxygenase (NCED), and Myo-inositol oxygenase (MIOX) have laid a foundation to predict the regulatory network of cotton genes in response to various abiotic stresses (Ma et al., 2016; He et al., 2017; Elasad et al., 2018; Shazadee et al., 2019; Chen et al., 2020; Li Z. et al., 2020; Wang W. et al., 2020; Zhao et al., 2020; Li Q. et al., 2021). To gain a better understanding, we summarized the regulatory networks of cotton genes in response to salt and drought in this review (Table 1).

**TABLE 1:** Summary of cotton genes regulatory networks in response to salt and drought.

**Functional category**	**List of genes**	**Type of stress**	**Signaling pathway**	**References**
Protein kinase				
MAPKKK	*GhMAP3K40, GhMAPKKK49*	Abiotic and biotic stress	ABA	Chen X. et al., 2015; Na et al., 2019; Nadarajah, 2020
	*GhRaf19*	Salt and drought	NA	Jia et al., 2016
	*GhRAF4* and *GhMEKK12*	Drought	NA	Zhang et al., 2020
MAPKK	*GhMKK1*	Influences oxidative, salt and drought	SA	Nadarajah, 2020; Wang G. et al., 2020
	*GhMKK3*	Influences oxidative, salt, and drought stresses	SA	
	*GhMKK4, GhMKK5*	Influences oxidative, drought	JA	
	*GhMKK9*	Salt and/or drought	ET	
MAPK	*GhMPK7*	Influences oxidative, Salt, drought	JA, SA	Shi et al., 2010
	*GbMPK3*	Drought	NA	Long et al., 2014
	*GhMPK3*	Cold, Drought, and Salt	NA	Sadau et al., 2021
	*GhMPK17*	Influences oxidative, Salt, drought	JA	Zhang J. et al., 2014
Transcription factor				
bZIP	*GhABF2*	Salt, drought	ABA	Liang et al., 2016
	*ABP9*	Salt, drought	ABA	Wang C. et al., 2017
bHLH	*GhbHLH1*	drought	ABA	Meng et al., 2009
NAC	*GhirNAC2*	Drought	ABA	Shang et al., 2020
	*GhATAF1*	Salt	SA	He et al., 2016
	*GhNAC18*	Drought	SA, JA, and ET	Evans et al., 2016
ERF/DREB	*GhDREB1L*	Drought and high salinity	NA	Huang et al., 2007
	*GhERF2, GhDREB1B, and GhDREB1A*	Drought	NA	Baillo et al., 2019
	*GhERF38*	Salt, drought	ABA	Ma et al., 2017
	*GhDREB40D and GhDREB7A*	Drought	NA	Debbarma et al., 2019
MYB	*GhMYB73*	Salt	ABA	Zhao et al., 2019
	*GbMYB5*	Drought	ABA	Chen T. et al., 2015
	*GhMYB108*	Salt, drought	NA	Ullah et al., 2020
WRKY	*GhWRKY46*	Salt, drought	NA	Li Y. et al., 2021
	*GhWRKY27*	Drought	ABA	Yan et al., 2015
	*GhWRKY6*	Salt, drought	ABA	Li Z. et al., 2019
	*GhWRKY41*	Salt, drought	ABA	Chu et al., 2015
	*GhWRKY91, GhWRKY17*	Salt, drought	ABA	Gu et al., 2019
	*GhWRKY25*	Drought, salt	NA	Liu et al., 2016
	*GhWRKY33*	Drought	ABA	Wang N.N. et al., 2019
ROS-scavenging				
SOD	*GhSOD*	Salt	NA	Luo et al., 2013
APX	*GhAPX1*	oxidative	NA	Guo K. et al., 2016
POD	*GhPOD*	Salt	NA	Li S. et al., 2020
GST	*Gst-cr1*	Drought	NA	Yu et al., 2003
Ca^2+^ transporters and binding proteins	*GhCPK8*, *GhCPK38*, *GhCPK54*, and *GhCPK55*	Salt	NA	Gao et al., 2018
	*GhCIPK6a*	Salt	NA	Su et al., 2020
Others	*GhSOS1*	Salt	NA	Chen X. et al., 2017
	*GhNHX1*	Salt	NA	Long et al., 2020
	*GhPYL10*, *GhPYL12*, and *GhPYL26*	Drought	ABA	Chen Y. et al., 2017
	*Gh_A08G1120*	Salt, drought	NA	Kirungu et al., 2019
	*Gh_D06G0281*	Salt, drought, and cold	ABA	Lu et al., 2019
	*GhANN8b*	Salt	NA	Mu et al., 2019

*MAPKKK, mitogen-activated protein kinase kinase kinases; MAPKK, mitogen-activated protein kinase kinase; MAPK, mitogen-activated protein kinase; bZIP, basic leucine zipper; bHLH, basic helix–loop–helix; ERF/DREB, ethylene-responsive factor/dehydration-responsive element-binding; MYB, myeloblastosis; SOD, superoxide dismutases; APX, ascorbate peroxidase; POD, peroxidase; GST, glutathione S-transferase; ABA, abscisic acid; SA, salicylic acid; JA, jasmonic acid; ET, ethylene; BR, brassinolide; NA, not available.*

## Genes Involved in Response to Wilt Disease (*Verticillium* and *Fusarium*)

Plants can develop resistance to *Verticillium* and *Fusarium* through a variety of mechanisms, including cell wall modifications, extracellular enzymes, pattern recognition receptors, TFs, and signal transduction pathways related to SA/JA/ET (Song et al., 2020). Several studies have been conducted over the last decade on the physiological and molecular mechanisms of plant resistance to *V. dahliae* and *F. oxysporum* wilt. Many resistance-related genes are summarized in this review to provide a theoretical foundation for a better understanding of the molecular genetic mechanisms underlying plant resistance to *Verticillium* and *Fusarium* wilt disease (Table 2). Moreover, this review is intended to be a resource for future research on the development of genetic resistance mechanisms to combat fungal wilt diseases.

**TABLE 2:** Summary of cotton genes regulatory networks in response to *Verticillium* and *Fusarium* wilt disease.

**Functional category**	**List of genes**	**Type of stress**	**Signaling Hormone**	**References**
Signaling transduction	*GhMPK20*	*F. oxysporum*	SA	Wang et al., 2018
	*GhMKK4*	*F. oxysporum*	SA	Wang et al., 2018
	*GhMKK4, GhMKK6*, and *GhMKK9*	*V. dahliae*	NA	Meng et al., 2018
	*GhMORG1*	*F. oxysporum*	NA	Wang C. et al., 2020
	*GaRPL18*	*V. dahliae*	SA	Gong et al., 2017
	*GaGSTF9*	*V. dahliae*	SA	Gong et al., 2018
	*GhCPK33*	*V. dahliae*	JA	Hu et al., 2018
	*GbERF1*	*V. dahliae*	ET	Guo W. et al., 2016
	*GbABR1*	*V. dahliae*	ET	Liu Y. et al., 2018
	G*hSAMDC*	*V. dahliae*	SA	Mo et al., 2016
	*GbaNA1*	*V. dahliae*	SA, JA, ET	Li et al., 2018a
	*GhTGA7* and *GhBZR1*	*V. dahliae*	SA, BR	Zhang et al., 2016
	*GhNDR1* and *GhMKK2*	*V. dahliae*	NA	Gao et al., 2011
Transcription factor	*MYB46* and *MYB86*	*V. dahliae*	NA	Zhu et al., 2021
	*GhMYB108*	*V. dahliae*	NA	Cheng et al., 2016
	*GbVIP1*	*V. dahliae*	ET	Zhang K. et al., 2019
	*HDTF1*	*V. dahliae*	JA	Gao et al., 2016
	*GbWRKY1*	*V. dahliae*	JA	Li et al., 2014
	*GbERFb*	*V. dahliae*	ET	Liu J. et al., 2017
	*GbNAC1*	*V. dahliae*	NA	Wang et al., 2016b
	*GhBLH7-D06*	*V. dahliae*	JA	Ma et al., 2020
	*GhHB12*	*V. dahliae*	JA	He et al., 2018
Defense-related proteins	*GhPLP2*	*V. dahliae*, *F. oxysporum*	ET, JA	Zhu et al., 2021
	*GhPGIP1*	*V. dahliae*	NA	Liu N. et al., 2017
	*GbNRX1*	*V. dahliae*	NA	Li et al., 2016
	*GbHyPRP1*	*V. dahliae*	NA	Yang J. et al., 2018
	*GhGLP2*	*V. dahliae*, *F. oxysporum*	NA	Pei et al., 2020
	*GhMLP28*	*V. dahliae*	JA, SA, and ET	Shi et al., 2012
	*GhUMC1*	*V. dahliae*	JA	Zhu et al., 2018
	*GhTLP19*	*V. dahliae*	NA	Wang et al., 2020a
	*GbEDS1*	*V. dahliae*	SA	Yan et al., 2016
	*GhRD21-7*	*V. dahliae*	NA	Li R. et al., 2019
	*GhBOP1*	*V. dahliae*	NA	Zhang Z. et al., 2019
Cellular enzymes	*Chi23*, *Chi32*, or *Chi47*	*V. dahliae*	NA	Xu et al., 2016
	*Chi28*	*V. dahliae*	NA	Han et al., 2019
	*Lyp1*, *Lyk7*, and *LysMe3*	*V. dahliae*	JA, SA	Xu et al., 2017
	*GhPMEI3*	*V. dahliae*	NA	Liu N. et al., 2018
	*caffeic acid 3-O-methyltransferase* and *peroxidase2*	*F. oxysporum*	NA	Hou et al., 2021
	*GhLAC15*	*V. dahliae*	NA	Zhang Y. et al., 2019
	*GhUMC1*	*V. dahliae*	JA	Zhu et al., 2018
	*GhWAT123*	*V. dahliae*	NA	Tang et al., 2019
	*Gh4CL30*	*V. dahliae*	NA	Xiong et al., 2021
	*GhECR*	*F. oxysporum, V. dahliae*	NA	Mustafa et al., 2017
	*GbSBT1*	*V. dahliae*	JA, ET	Duan et al., 2016
Receptor like and other proteins	*GhRLPGSO1*-like, *GhRLP44*, GhRLP6, and *GhRLP34*	*F. oxysporum*	NA	Cilkiz, 2017
	*GbRLK*	*V. dahliae*	NA	Jun et al., 2015
	*GhlncNAT-ANX2* and *GhlncNAT-RLP7*	*V. dahliae*	NA	Zhang et al., 2018
	*GbAt11*	*V. dahliae*	NA	Qiu et al., 2020
	*GbCAD1*	*V. dahliae*	NA	Gao et al., 2013
	*GbANS*	*V. dahliae*	NA	Long et al., 2018

### Signaling Transduction

A series of complex signal transduction processes and phytohormones function directly to control plant immunity systems. Amongst the intricate signaling networks, MAPK cascades are the primary modules responsible for classifying and amplifying external signals into intracellular components (Meng and Zhang, 2013). They are significant in both biotic and abiotic stress (Shi et al., 2011). Different signal transduction pathways operate independently while also exhibiting significant crosstalk (Gull et al., 2019). It complicates their comprehension of biotic stimuli. Multiple genes that are affected by biotic stresses suggest that there may not be a single stress tolerance marker.

Expression of *GhMPK20* is significantly induced by *F. oxysporum*. *GhMPK20* silencing in cotton increased tolerance to *F. oxysporum*, whereas ectopic *GhMPK20* overexpression in tobacco decreased *F. oxysporum* resistance by interfering with the SA-mediated defense pathway. Moreover, *GhMKK4* and *GhWRKY40* silencing improved F. oxysporum resistance in cotton, and GhMKK4-GhMPK20 function was revealed to be required for *F. oxysporum*-induced *GhWRKY40* expression (Wang et al., 2018). More importantly, using gene silencing techniques, *GhNDR1* and *GhMKK2* have been shown to be essential for *Verticillium* resistance in cotton (Gao et al., 2011). MKK members in MAPK signaling cascades also play dual functions in delicately modulating cotton plant resistance to fungal wilt; *GhMKK4*, *GhMKK6*, and *GhMKK9* positively regulate cotton *Verticillium* resistance, while *GhMKK10* negatively regulates it (Meng et al., 2018). Recently, a cotton MAPK scaffold protein (*GhMORG1*) was shown to interact with *GhMKK6* and GhMPK4, and the overexpression of *GhMORG1* in cotton protoplasts significantly increased the activity of the GhMKK6-GhMPK4 cascade that positively regulates the resistance of cotton to *F. oxysporum* (Wang C. et al., 2020).

The SA-mediated glutathione S-transferase *GaGSTF9* was a positive regulator to *Verticillium* wilt based on VIGS and overexpression in *Arabidopsis* (Gong et al., 2018). Expression of the ribosomal protein, *GaRPL18*, is induced by SA treatment, suggesting an association of *GaRPL18* in the SA signal transduction pathway. Importantly, due to a considerable decrease in the amount of immune-related molecules, wilt-resistant cotton species in which *GaRPL18* was silenced became more susceptible to *V. dahliae* than control plants. In contrast, overexpressing *GaRPL18* resulted in more resistance to *V. dahliae* infections (Gong et al., 2017). Expression of cyclin-dependent kinase E (*GhCDKE*) in cotton was induced by *V. dahliae* infection and MeJA treatment, and silencing of *GhCDKE* led to enhanced susceptibility to *V. dahliae* in cotton, while overexpression of *GhCDKE* improved resistance to this pathogen in *Arabidopsis* (Li et al., 2018). A calcium-dependent protein kinase, *GhCPK33*, derived from upland cotton functions as a negative regulator of *V. dahliae* resistance, which is induced by JA biosynthesis. Knockdown of *GhCPK33* enhanced resistance to *V*. *dahliae* (Hu et al., 2018).

Knock-down of SA-related Spermine (Spm) proteins, Spm synthase (GhSPMS), and S-adenosylmethionine decarboxylase (*GhSAMDC*), damages plant resistance to *V. dahliae* infection in cotton. In contrast, enhanced resistance to transgenic *Arabidopsis* suggests that *GhSAMDC* contributes in plant resistance to *V. dahliae via* SA and leucine-related signaling pathways and mediates Spm biosynthesis (Mo et al., 2016). *GhPAO* expression in Arabidopsis improves resistance to *V. dahliae* and affects the accumulation of high levels of H_2_O_2_, SA, and camalexin (a phytoalexin), implying that GhPAO contributes to plant resistance to *V. dahliae* by activating Spm and camalexin signaling pathways (Mo et al., 2015). Walls are thin (*WAT*) promotes the resistance of crops to a wide range of pathogens by regulating SA metabolism and signaling transduction through affecting the polar transport of auxin (Denancé et al., 2013). *GhWATs* knockdown increased SA content accumulation, triggered SA pathway-related gene expression, and increased lignin accumulation in xylem sections, all of which accelerated plant resistance to *Verticillium* wilt (Tang et al., 2019).

Ethylene-responsive factors (ERFs) are generally required for pathogen defense responses. However, only a few ERF genes have been characterized in cotton in response to fungal wilt. *GbABR1* is a member of the AP2 family and an ERF subfamily B4 member from *G. barbadense*. Silencing *GbABR1* in cotton plants resulted in a higher disease index, showing that this gene positively contributes to *Verticillium* wilt resistance (Liu Y. et al., 2018). *GbERF1*-like, ET response-related factor contributes to plant resistance against *V. dahliae* by positively regulating lignin synthesis (Guo W. et al., 2016). Nucleotide-binding site leucine-rich repeat (NBS-LRR) proteins play an important role in plant defense against fungal pathogens. A NBS-LRR gene *GbaNA1* derived from *Gossypium barbadense* can be induced by *V. dahliae* and by the phytohormones SA, ET, and JA participating in island cotton resistance to *V. dahliae* (Li et al., 2018b). Prominently, overexpression of *GbaNA1* increases ROS content in *Arabidopsis* and the expression of genes associated with the ET signaling pathway (Li et al., 2018a). Additionally, eight differentially expressed candidate genes in SA (*GhPUB17*, *GhTGA7*, and *GhPR1*), JA (*GhJAZ10* and *GhbHLH18*), ET (*GhEBF1*), cytokinine (*GhE13L13*), and BR (*GhBZR1*) signal pathways were investigated using VIGS techniques in the transcriptome with *V. dahliae* infection and non-infection. Knock-down of up-regulated genes *GhJAZ10, GhPUB17*, *GhbHLH18*, and *GhEBF1* significantly enhanced susceptibility of resistant varieties to *V. dahliae*, while silencing down-regulated genes *GhTGA7* and *GhBZR1* significantly improved resistance of susceptible varieties to *V. dahliae* (Zhang et al., 2016). This revealed that genes from different hormone signaling pathways have important roles in response to fungal wilt infection.

### Transcription Factors

Plant TF responses to biotic stress are extremely complex, with several TF families clearly linked to single or multiple stresses, as well as complex cross-talk between different signal transduction pathways. Proteins of the MYB family function as TFs involved in defense against pathogen infection. Two TFs, *MYB46* and *MYB86*, are probably involved in the accumulation and synthesis of lignin suggesting that they can be used to detect *Fusarium* wilt resistant cotton (Zhu et al., 2021). Knockdown of *GhMYB108* expression conferred enhanced susceptibility of cotton plants to *V. dahliae*, whereas overexpression of *GhMYB108* in *Arabidopsis* led to improved tolerance (Cheng et al., 2016). Silencing of a home domain transcription factor gene (*HDTF1*) derived from cotton significantly improved cotton plant resistance to *V. dahliae via* activation of the JA-mediated signaling pathway (Gao et al., 2016). A stress-responsive HD-ZIP ∣ TF *GhHB12* in cotton was induced by JA and *V. dahliae* infection, and cotton plant susceptibility to the fungal pathogens *Botrytis cinerea* and *V. dahliae* was increased by overexpression of *GhHB12*, which was coupled with suppression of the JA-response genes *GhJAZ2* and *GhPR3* (He et al., 2018). bHLH is another TF that functions against plant pathogens. As such, *GbbHLH171* cooperates with and is phosphorylated by a defense-related receptor-like kinase (*GbSOBIR1*) in *G. barbadense*, and had a positive role on cotton resistance to *V. dahliae* (Zhou et al., 2019). *GbWRKY1* is a key regulator that mediates the plant defense-to-development transition by activating JAZ1 expression during *V. dahliae* infection, and it has been shown to be a negative regulator of the JA-mediated defense pathway, participating in plant resistance against *V. dahliae* and *B. cinerea* (Li et al., 2014).

*GbVIP1* (VirE2 interaction protein 1), which encodes a bZIP TF protein, was cloned in *G. barbadense*. Inoculation with *V. dahliae* and exogenous ET treatment both increased GbVIP1 expression. VIGS showed that silencing of *GbVIP1* decreased cotton resistance to *Verticillium* wilt, while ectopic expression of *GbVIP1* in tobacco improved resistance to *Verticillium* wilt by up-regulating *PR1*, *PR1-like*, and *HSP70* genes (Zhang K. et al., 2019). The BEL1-Like TF *GhBLH7-D06*, which is commonly expressed in vascular tissues, functions in formation of secondary cells and also responds to *V. dahliae* infection, is induced by phytohormone JA treatment. The loss of function expression of *GhBLH7-D06* could increase the resistance of cotton plants against *Verticillium* wilt. This resistance may be primarily due to the notable overexpression of genes involved in lignin biosynthesis and the JA signaling pathway, which also suggests that *GhBLH7-D06* negatively controls cotton resistance to *Verticillium* wilt (Ma et al., 2020). Additionally, knock-down of *GbNAC1* TFs showed that cotton was susceptible to *Verticillium* wilt, and *GbNAC1*-overexpressed in transgenic *Arabidopsis* plants enhanced resistance to *V. dahliae* compared to wild type (Wang et al., 2016b). *GbERFb*, a AP2/ERF type TF, can also improve cotton disease resistance (Liu J. et al., 2017). The findings from previous reports suggest that TF genes could be used to improve biotic stress tolerance/resistance in important cotton crops; though, more research is needed to understand the mechanisms of these TFs.

### Defense-Related Proteins

Plants’ resistance to fungal pathogens is greatly influenced by defense-related proteins. Plants have evolved intricate sensory mechanisms to detect biotic invasion and overcome the negative effects on growth, yield, and survival (Iqbal et al., 2021). Therefore, plants have evolved a plethora of responses to defend themselves against a wide range of pests and pathogens. Patatin-like proteins (PLPs) are defensive proteins with non-specific lipid acyl hydrolyze activity, which can hydrolyze membrane lipids into fatty acids and lysophospholipids. The importance of PLPs in plant growth and abiotic stress has been extensively studied (Jenks and Wood, 2009; Kim et al., 2014; Cheng et al., 2019; Gao et al., 2021), but the molecular function of PLPs in the plant defense system against fungal wilt is still poorly known.

*GhPLP2*, a cotton PLP protein located in the cell wall and plasma membrane was highly induced by treatment with *V. dahliae*, *F. oxysporum*, and signaling molecules ET and JA in cotton plants. Silence of *GhPLP2* cotton plants showed reduced resistance to *V. dahliae* infection, whereas overexpression of *GhPLP2* in *Arabidopsis* enhanced resistance to *V. dahliae*, with mild symptoms and lower disease index and fungal biomass. Moreover, GhPLP2-transgenic plants had higher accumulation of JA and JA synthesis precursor linoleic acid and α-linolenic acid than control plants, showing that PLPs have a positive role against fungal pathogenicity and have a significant role in the pathogenicity of *V. dahliae* (Zhu et al., 2021). Overexpression of *CkPGIP1* from *Cynanchum komarovii* and *GhPGIP1* from *G. hirsutum* can improve cotton resistance to *V. dahliae* by increasing the expression of pathogenesis-related proteins (PRs) and increasing disease susceptibility, as well as phytoalexin-deficient and isochorismate synthase genes that upregulate xylem lignification (Liu N. et al., 2017). *GbNRX1* is an apoplastic thioredoxin protein found in *Verticillium* wilt-resistant island cotton, which is connected to an increase in abundance in response to infection with *V. dahliae*. The higher accumulation of ROS in apoplastic and reduced *V. dahliae* resistance in GbNRX1-silenced plants show that *GbNRX1* can improve immune response against this fungus (Li et al., 2016).

BLADE-ON-PETIOLE1 (BOP1) and BOP2 are two BTB-ankyrin proteins that are specifically expressed in lateral-organ boundaries (LOBs). Silencing and overexpression studies show that *GhBOP1* is a positive regulator of plant resistance to *V. dahliae*. Moreover, *GhBOP1* works in tandem with *GhBP1* to modulate lignin biosynthesis, conferring enhanced resistance to *V. dahliae* in cotton plants (Zhang Z. et al., 2019). Cotton *GbHyPRP1* encodes a protein with both proline-rich repetitive and pollen ole e I domains. Cotton resistance to *V. dahliae* was improved in HyPRP1-silent plants through cell wall thickening and ROS accumulation. Overexpression of *HyPRP1* in transgenic *Arabidopsis* plants significantly enhanced resistance to *V. dahliae* (Yang J. et al., 2018).

A defense-related major latex protein (*GhMLP28*) derived from upland cotton was induced by *V. dahliae* infection, JA, SA, and ET treatment. Knock-down of *GhMLP28* increases cotton plant susceptibility to *V. dahliae* infection, while *GhMLP28* ectopic overexpression improves disease resistance in tobacco. *GhDIR1* encodes a putative dirigent protein, and its overexpression increases lignin content in transgenic cotton plants, resulting in increased tolerance to *V. dahliae* infection (Shi et al., 2012). Both gain and loss of function analyses revealed that *GhUMC1*, a cotton umecyanin-like gene, is involved in *V. dahliae* resistance *via* regulation of the JA signaling pathway and lignin metabolism (Zhu et al., 2018). The defense regulator enhanced disease susceptibility 1 (EDS1), encoding a lipase-like protein induced by SA, has a crucial role against pathogens. *GbEDS1*, overexpression in Arabidopsis increased SA and H_2_O_2_ production, leading to increased disease resistance to *V. dahliae*. GbEDS1-silencing in *G. barbadense* significantly reduced SA and H_2_O_2_ accumulation, resulting in increased susceptibility (Yan et al., 2016).

Moreover, papain-like cysteine proteases (PLCPs), a large plant family, are thought to play a role in plant defense against pathogens. Transcriptome analysis revealed that *GhRD21-7* genes in cotton were significantly up-regulated in response to *V. dahliae*. More importantly, over-expression of *GhRD21-7* improved resistance, while RNAi lines were more susceptible to *V. dahliae* in cotton (Li R. et al., 2019). Recently, germin-like proteins (GLPs), a diverse and ubiquitous family of plant glycoproteins were identified as part of the cupin super family; they have notable roles in plant defense against various abiotic and biotic stresses. Silencing of *GhGLP2* in upland cotton enhanced susceptibility to *V. dahliae* and *F. oxysporum* with resulting severe wilt on leaves, enhanced vascular browning, and inhibited callose deposition. Overexpression in *Arabidopsis* exhibited significant resistance to *V. dahliae* and *F. oxysporum*, with decreased mycelial growth, increased callose deposition and cell wall lignification at infection sites on leaves (Pei et al., 2020). Thaumatin-like proteins (TLPs), another type of defense related protein (PR-5) in a large multigene family, have important roles in biotic and abiotic stress. When *GhTLP19* was silenced in cotton the plants were more sensitive to *V*. *dahliae*, with increased MDA content and decreased CAT content, and as well as increased disease index (DI) and hyphae accumulation (Wang et al., 2020a). These studies describe cotton defense-related proteins, as well as their putative mechanisms of action, pathogen targets, and biotechnological implications.

### Cellular-Bound Enzymes

Extracellular enzymes in plants are the first line of protection against fungal pathogens. The available research supports the fact that extracellular enzyme is not only a part of the adaptations that help cotton plants cope with pathogen infections, but it is also a key metabolic enzyme that enhances cotton plants’ growth and development. Many studies have shown that chitinase (Chi) has a housekeeping role in plasticizing the cell wall as a key hydrolytic enzyme, which destroys the fungal cell wall (Xu et al., 2016). Moreover, *Chi* expression can be instigated in response to biotic and abiotic stress in plants (Cheng et al., 2017). A total of 47, 49, 92, and 116 *Chi*s from four sequenced cotton species, diploid *G. raimondii* and *G. arboreum* and tetraploid *G. hirsutum* and *G. barbadense* were, respectively identified. Phylogenetic classification classified these *Chi*s into six groups. Cotton resistance to *V. dahliae* was significantly reduced when *Chi23*, *Chi32*, or *Chi47* genes were knockdown, indicating that these genes function as positive regulators of *V. dahliae* (Xu et al., 2016). *Chi28* includes the class IV *chitinase* subfamily. Silencing of *CRR1* or *Chi28* led to cotton plants more susceptible to *V. dahliae* infection, while overexpression of *CRR1* increased *V. dahliae* resistance (Han et al., 2019). In plants, lysin motif (LysM)-containing proteins play a key role in chitin recognition as well as the control of defense responses against fungal pathogen attack. The genes *Lyp1*, *Lyk7*, and *LysMe3* were found in the plasma membrane, and knockdown of their expression in cotton significantly reduced SA, JA, and ROS generation, decreased defense gene activation, and negotiated resistance to *V. dahliae* (Xu et al., 2017).

*Pectins* are the major elements of the primary plant cell wall, and play an important role in pathogen defense mechanisms. Pectin methylesterases (PMEs) have protective roles in the plant cell wall through catalyze dimethyl esterification of the homogalacturonan domains of pectin. Silencing *GhPMEI3* in upland cotton results in increased susceptibility to *V. dahliae* infection, while ectopic expression of *GhPMEI3* increased pectin methyl esterification and limited fungal disease by modulating root elongation. In addition, *GhPMEI3* and *GhPME* may be involved in protein-protein interactions and are important for plant evolution to resist fungal diseases (Liu N. et al., 2018). Furthermore, erasure of two pectin lyase genes (*VdPL3.1* and *VdPL3.3*) reduced wilt virulence to cotton. This study shows that the *V. dahliae* exoproteome plays an important role in the development of wilting and necrosis symptoms, primarily through pathogenic mechanisms of plant cell wall degradation as part of host plant infection (Chen J.Y. et al., 2016). Cotton resistance to fungal wild diseases is largely determined by lignin synthesis. The resistant cotton cultivars accumulate a significant amount of lignin and lignin-like phenolic polymers. Increasing evidence shows that lignin content is positively correlated with resistance to fungal wilt (Xu et al., 2011). Two coding genes in cotton, *caffeic acid 3-O-methyltransferase* and *peroxidase2*, are probably involved in the accumulation and synthesis of lignin in response to *Fusarium* wilt (Hou et al., 2021). Hence, quantification of lignin can be used as a selection tool to identify *Fusarium* resistant cotton.

A laccase gene, *GhLAC15*, has been found to be highly inducible by pathogens. Additionally, Transgenic expression enhances *Verticillium* wilt resistance by increasing defense-induced lignification, arabinose, and xylose accumulation in the cotton cell wall (Zhang Y. et al., 2019). *GhUMC1*, a blue copper-binding protein, is involved in cotton resistance to *V. dahliae via* lignin synthesis and in cell wall remodeling through the JA signaling pathway (Zhu et al., 2018). Three concurrently silenced *GhWATs* (*GhWAT123*-silenced), repressed plant growth and increased plant resistance to *V. dahliae* by increasing lignin deposition in the xylem (Tang et al., 2019). Knockdown of cotton lignin biosynthetic gene *Gh4CL30* led to decreased content of flavonoids, lignin, and S monomer but an increased content of G monomer, G/S lignin monomer, caffeic acid, and ferulic acid, providing new insights into cotton resistance to *V. dahliae* (Xiong et al., 2021).

The cotton enoyl-CoA reductase (GhECR) gene functions directly in very-long-chain fatty acid formation. VIGS analysis exposed that GhECR-silenced plants are more sensitive to *V. dahliae* and *F. oxysporum* infection, showing that the *GhECR* gene is linked to cotton resistance to various *V. dahliae* and *F. oxysporum* strains (Mustafa et al., 2017). *GbSBT1*, a subtilase like protein derived from *G. barbadense* located on the cell membrane, is highly induced by *V. dahliae*, JA, and ET treatment. Moreover, silencing the GbSBT1 gene decreases the tolerance to *V. dahliae* infection. Notably, in *Arabidopsis*, overexpression of *GbSBT1* stimulates the expression of defense-related genes and enhances resistance to *F. oxysporum* and *V. dahliae* (Duan et al., 2016). These findings revealed that the induced defensive enzymes, which are produced in response to an attack, provide remarkable protection against pathogens *via* defense mechanisms.

### Receptor Like and Other Proteins

Plant receptor-like proteins are involved in diverse of biological processes, including development, innate immunity, cell differentiation and patterning, nodulation, and self-incompatibility (Yang et al., 2012). Receptor-like proteins are on the front lines of the plant-pathogen battle because they are present at the plasma membrane and detect signature molecules from either the invading pathogen or damaged plant tissue. Cell-surface-associated PRRs are essential in fungal pathogen recognition. PRRs are receptor-like kinases (RLKs) and receptor-like proteins (RLPs) found in plants. Using the gene silencing approach, it was suggested that *GhRLPGSO1*-like, *GhRLP44*, GhRLP6, and GhRLP34 might be needed for defense against *F. oxysporum* in cotton plants (Cilkiz, 2017). An RLK gene (*GbRLK*) from the disease-resistant cotton *G. barbadense*, is stimulated with the infection of *V. dahliae*. In addition, transgenic cotton and Arabidopsis plants of *GbRLK* confer resistance to *V. dahliae* infection (Jun et al., 2015). NBS-LRR (nucleotide-binding site leucine-rich repeats) proteins play an important role in plant pathogen defense. A genome-wide association study revealed that TIR-NBS-LRR domains containing CG02 are the most likely candidate associated to cotton resistance against *V. dahliae*. According to Real-time quantitative PCR and VIGS analysis, CG02 was specific to up-regulation in the resistant genotype, and silenced plants were more susceptible to *V. dahliae* (Li T. et al., 2017).

Furthermore, the Ve R-gene locus contributes to *Verticillium* resistance by encoding RLPs with extracellular leucine-rich repeats (Nazar et al., 2018). Several studies explored how Ve1 is involved in the cotton resistance to *Verticillium* wilt infection (Zhang B. et al., 2012; Chen T. et al., 2016; Song et al., 2017, 2020; Yang Y. et al., 2018). *GhlncNAT-ANX2 and GhlncNAT-RLP7* are conserved long non-coding RNAs, and their silencing in cotton promotes resistance to *V. dahliae*, which may be connected to the upregulated expression of *lipoxygenase 1* and *lipoxygenase 2* (Zhang et al., 2018). *GbAt11* (AXMN Toxin Induced Protein-11) was found to be highly resistant to *Verticillium* wilt in *G. barbadense*. Moreover, FLS2, BAK1, and other disease resistance genes can be up-regulated by *GbAt11* overexpression (Qiu et al., 2020). *GhPUB17*, a U-box E3 ubiquitin ligase that interacts with and is inhibited by the antifungal protein *GhCyP3*, negatively regulates cotton resistance to the *Verticillium* wilt pathogen (Qin et al., 2019). Moreover, anthocyanin and production of gossypol is sufficient to influence *V. dahliae* infection. As such, cotton *GbANS* is involved in anthocyanin biosynthesis, and silencing *GbANS* significantly decreases anthocyanin production as well as cotton plant tolerance to *V. dahliae* (Long et al., 2018). Silencing of *GbCAD1*, which encodes a key enzyme contributed in gossypol biosynthesis, compromises cotton plant resistance to *V. dahliae* (Gao et al., 2013).

## Future Perspectives and Conclusion

Due to increasing incidences of both biotic and abiotic stresses, sustainability of crop production is a serious challenge under field conditions. The cotton mechanisms that exhibit tolerance to various biotic and abiotic stresses appear to be interrelated and may have overlapping genetic elements. Both abiotic and biotic stresses negatively affect molecular, biochemical, and physiological processes, ultimately resulting in suppressed growth and development in cotton, such as reduced photosynthetic rate, plant height, leaf and root size, biomass, yield, and yield components, and poorer fiber quality. Currently, many phytohormone-based growth regulators are commercially used in agriculture to improve the resistance of plants to abiotic and biotic stresses. This indicates that identification and characterization of genetic components such as defense of cell membranes and proteins, signaling cascades and transcriptional control, and ion uptake and transport and their relevant biochemical pathways and multiple signal factors, are necessary to provide important clues to understand basic molecular mechanism/network of plant response and the development of plants with better resistance to adverse conditions. Nevertheless, due to the complexity of stress conditions and the difficulty of phenotyping, the genetic basis of this tolerance is not fully understood, because it is affected by multiple gene regulatory systems with environmental influences. However, drought alone impacts 45% of the world’s agricultural land; additionally, 19.5% of irrigated agricultural lands are classified as saline (Abdelraheem et al., 2019). A combination of two or more abiotic stresses, such as drought and salinity, results in greater yield loss than either stress alone. Drought and salinization are expected to cause up to 50% of arable land loss globally. Moreover, *Verticillium* and *fusarium* wilt are caused by soil-borne pathogenic fungi, and are major constraints to cotton production (Pei et al., 2020). Therefore, one of the most practical solutions is the development of abiotic (drought and/or salt) and biotic (*Verticillium* and *Fusarium*) stress tolerant cultivars. In recent decades, numerous genes responsive to drought, salt, and *Verticillium* and *Fusarium* wilt diseases in cotton have been identified, some of which were further studied using transgenic approaches, but none of the genes have been utilized in commercial cotton breeding programs. In this review, we summarized cotton genes related to salt, drought, and wilt disease resistance on the basis of their molecular functions. The review provides researchers with good theoretical knowledge and identifies gene networks that can help in discovering other resistance-related genes in order to better understand the molecular genetic mechanisms of cotton resistance to these stresses.

## Author Contributions

MB, FL, and ZY conceptualized the review. MB wrote the original draft. ZY and FL investigated, revised, and edited the draft manuscript. All authors contributed to the article and approved the submitted version.

## Conflict of Interest

The authors declare that the research was conducted in the absence of any commercial or financial relationships that could be construed as a potential conflict of interest.

## Publisher’s Note

All claims expressed in this article are solely those of the authors and do not necessarily represent those of their affiliated organizations, or those of the publisher, the editors and the reviewers. Any product that may be evaluated in this article, or claim that may be made by its manufacturer, is not guaranteed or endorsed by the publisher.
